# The estimation of additive genetic variance of body size in a wild passerine is sensitive to the method used to estimate relatedness among the individuals

**DOI:** 10.1002/ece3.10981

**Published:** 2024-02-13

**Authors:** Mónika Jablonszky, David Canal, Gergely Hegyi, Márton Herényi, Miklós Laczi, Gábor Markó, Gergely Nagy, Balázs Rosivall, Eszter Szöllősi, János Török, László Zsolt Garamszegi

**Affiliations:** ^1^ Evolutionary Ecology Research Group Institute of Ecology and Botany, HUN_REN Centre for Ecological Research Vácrátot Hungary; ^2^ Behavioural Ecology Group, Department of Systematic Zoology and Ecology ELTE Eötvös Loránd University Budapest Hungary; ^3^ Department of Evolutionary Ecology National Museum of Natural Sciences (MNCN‐CSIC) Madrid Spain; ^4^ Department of Zoology and Ecology Hungarian University of Agriculture and Life Sciences Godollo Hungary; ^5^ HUN‐REN‐ELTE‐MTM Integrative Ecology Research Group Budapest Hungary; ^6^ Department of Plant Pathology, Institute of Plant Protection Hungarian University of Agriculture and Life Sciences Budapest Hungary

**Keywords:** animal model, bird, evolution, quantitative genetics

## Abstract

Assessing additive genetic variance is a crucial step in predicting the evolutionary response of a target trait. However, the estimated genetic variance may be sensitive to the methodology used, e.g., the way relatedness is assessed among the individuals, especially in wild populations where social pedigrees can be inaccurate. To investigate this possibility, we investigated the additive genetic variance in tarsus length, a major proxy of skeletal body size in birds. The model species was the collared flycatcher (*Ficedula albicollis*), a socially monogamous but genetically polygamous migratory passerine. We used two relatedness matrices to estimate the genetic variance: (1) based solely on social links and (2) a genetic similarity matrix based on a large array of single‐nucleotide polymorphisms (SNPs). Depending on the relatedness matrix considered, we found moderate to high additive genetic variance and heritability estimates for tarsus length. In particular, the heritability estimates were higher when obtained with the genetic similarity matrix instead of the social pedigree. Our results confirm the potential for this crucial trait to respond to selection and highlight methodological concerns when calculating additive genetic variance and heritability in phenotypic traits. We conclude that using a social pedigree instead of a genetic similarity matrix to estimate relatedness among individuals in a genetically polygamous wild population may significantly deflate the estimates of additive genetic variation.

## Graphical abstract

Here, we compared different methods for estimating relatedness among the individuals (based on social pedigree or genomic data) to investigate additive genetic variance and heritability in the tarsus length of a passerine. Our results confirm the potential for this crucial trait to respond to selection and highlight methodological concerns when calculating additive genetic variance and heritability in phenotypic traits, as the heritability estimates were higher when obtained with the genetic similarity matrix instead of the social pedigree.

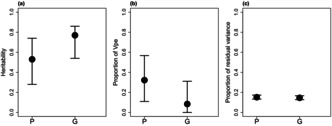

## 1 INTRODUCTION

Understanding the extent of additive genetic variation in phenotypic traits, particularly in wild populations, is essential to comprehend the processes of natural selection (Fisher, 1930; Mousseau & Roff, 1987). Assessing the capacity of populations to respond to selection is even more critical, in light of the ongoing rapid climate change (Merilä & Hendry, 2014; Møller et al., 2018; Okamiya et al., 2021), as climate change can increase directional selection and a lack of heritable variation could limit population persistence. Data to estimate additive genetic variance (*Va*) should be collected from wild populations, where natural evolutionary processes can be investigated. Unfortunately, collecting these data in wild populations is challenging (Pemberton, 2008; Postma, 2014). For example, separating genetic and environmental sources of variation is more challenging in wild populations than in laboratory or domesticated animals. This is primarily due to the higher environmental heterogeneity, smaller sample sizes, and confounding relations between environmental and genetic factors (that is, similarity is due to both genetic and environmental sources and their interaction) that generally characterize datasets collected from the wild (Bérénos et al., 2014; Kruuk & Hadfield, 2007). Furthermore, collecting data for pedigree construction is always time‐consuming as ideally it should cover several years (Quinn et al., 2006), and often it is not possible to assess kinship reliably based on observations in the wild. For example, due to extra‐pair copulations, the social pedigrees observed in the field do not always reflect the actual relatedness of the individuals (Alatalo et al., 1984; Pemberton, 2008).

Various approaches can be used to infer kinship or relatedness among individuals, which is a crucial step in estimating the *Va* of a trait. Historically, relatedness has been assessed based on social pedigrees (Pemberton, 2008), but, particularly in recent years, estimates based on genetic data are accumulating in the literature (Gienapp et al., 2017; Robinson et al., 2013; Visscher et al., 2008). This latter approach can be implemented by using pedigrees derived from genetic data or genetic relatedness matrices (Bérénos et al., 2014). Estimating relatedness based on genetic similarity is possible even without social pedigree information (Gienapp et al., 2017; Krag et al., 2013); consequently, it has now been attempted in many wild species, e.g., in mammals (Bérénos et al., 2014; Bonnet et al., 2022; Cristescu et al., 2022; Foroughirad et al., 2019; Gervais et al., 2019), birds (Bonnet et al., 2022; Robinson et al., 2013; Silva et al., 2017; Van Noordwijk et al., 1988), reptiles (Strickland et al., 2021), and fish (Garant et al., 2003; Reed et al., 2018). Assessing relatedness based on genetic data has several advantages compared to social pedigrees. For example, the risk of including erroneous links due to extra‐pair paternity (EPP) is much lower. Importantly, if relatedness is assessed based on markers, this should reflect the actual amount of the shared genome between the individuals (realized relatedness) instead of the expected relatedness that could be inferred from pedigrees (Visscher et al., 2006). Further, a genetic similarity matrix provides greater precision and higher information content, as it includes information on the relatedness among individuals whose relatives could not be determined (e.g., due to high dispersal or high population size relative to the sample) through the social pedigree (Perrier et al., 2018; Robinson et al., 2013). These are significant advantages, as it has been shown that the quality of the social pedigree (amount of missing or erroneous links) can bias the *Va* estimates, especially for complex models or when the true *Va* is low (Charmantier & Réale, 2005; Kruuk & Hadfield, 2007; Morrissey et al., 2007). On the other hand, social pedigrees may be the only means available to assess relatedness between individuals in some populations with long‐term historical data or when genetic data cannot be collected due to logistic or financial constraints. Social pedigrees can also reflect important information, as, for example, the quality of the social parents can influence trait expression (Lewis et al., 2021; Szöllősi et al., 2009). During the collection of data for a social pedigree, it is also easy to collect other relevant data such as the age, sex, survival, and health status of the individuals and observed pedigrees can also be a useful tools during quantitative analysis like linkage mapping or genome‐wide association studies (Galla et al., 2022). Furthermore, some critiques exist on estimating heritability based on genetic markers (de los Campos et al., 2015; Pemberton, 2008). Specifically, heritability could be biased if linkage disequilibrium is ignored as linkage disequilibrium patterns between markers can be very different in close and distant relatives (de los Campos et al., 2015) or if a large part of the variance is caused by many rare variants missing from the markers (Zaitlen et al., 2013). However, genetic similarity matrices based on single‐nucleotide polymorphisms (SNPs) have been shown to be appropriate for estimating *Va* when related individuals are adequately accounted for statistically and a large number of SNPs are used (usually >10,000; Bérénos et al., 2014; Lee & Chow, 2014; Purcell et al., 2007; Widmer et al., 2014). As both social pedigree‐ and genetic information‐based approaches have potential advantages, it is worthwhile to compare the results obtained based on social pedigrees versus genetic information and to examine the potential causes of the differences, e.g., the effect of erroneous pedigree links. However, to our knowledge, this comparison has rarely been conducted in wild populations (Bérénos et al., 2014; Perrier et al., 2018; Robinson et al., 2013).

Body size undoubtedly has an essential evolutionary significance (Bourne et al., 2022; Debes et al., 2021; Møller et al., 2018). It is related to fitness in many taxa, from arthropods to birds, through, for example, juvenile survival (Alatalo & Lundberg, 1986), longevity (Grant & Grant, 2000), susceptibility to predation risk (Götmark & Post, 1996; Lima, 1993; Møller & Nielsen, 2006), dominance (Brown & Maurer, 1986; Garnett, 1981; Han et al., 2015), and reproductive success (Alatalo & Lundberg, 1986; Canal et al., 2011; Lessard et al., 2014). Body size is also associated with various fitness‐related behaviors, such as mate choice (Alatalo & Lundberg, 1986), singing (Bueno‐Enciso et al., 2016; Kagawa & Soma, 2013; Linhart & Fuchs, 2015), risk‐taking behavior (Näslund et al., 2015), and foraging (Schröder et al., 2016; Wright et al., 2015). As in other traits, selection pressure on body size may fluctuate over time. In particular, due to warm weather conditions, animals are expected to become smaller according to Bergmann's rule, which has been confirmed in a wide range of taxa (Bourne et al., 2022; Caruso et al., 2014; Okamiya et al., 2021; but see: Salewski et al., 2014; Sheridan et al., 2018). However, to what extent these responses are caused by genetic evolution or environmental effects is not fully known in natural populations, although several studies presented data on *Va* in wild populations (Christe et al., 2000; Husby et al., 2011; Perrier et al., 2018; Van Noordwijk et al., 1988). However, more studies are needed, as comparing patterns among populations and species is essential to understand body size evolution under natural conditions comprehensively (Caruso et al., 2014; Merilä & Hendry, 2014).

In this field study, we investigated the *Va* of tarsus length, an easily quantifiable and widely used skeletal trait reflecting the full body size in birds, using a long‐term dataset from collared flycatchers (*Ficedula albicollis*). Our aim was twofold: (i) to shed light on the reliability of using social pedigree rather than genetic similarity matrices when estimating relatedness among individuals and on how the use of different methods affects the estimates of *Va* and (ii) to increase our knowledge on the *Va* in body size in wild bird populations. Moderate to high heritability estimates were reported for this species in other, remote populations (Merilä & Gustafsson, 1996; Silva et al., 2017), but the study population has not yet been investigated. We compared different approaches to estimate the heritability. Specifically, we used two matrices to determine the relatedness among individuals: one based on social pedigree and one based on genetic data. We also assessed the correlation between the two matrices and calculated the EPP rate (hereafter the proportion of chicks from extra‐pair paternity). Based on previous work in this species (Merilä & Gustafsson, 1996), we expected high *Va* in tarsus length. We predicted lower estimates when using the social pedigree instead of the genetic similarity matrix due to the moderate level of EPP reported in the study population (Garamszegi et al., 2004; Garamszegi & Møller, 2004; Rosivall et al., 2009).

## 2 METHODS

### 2.1 Study site and species

The collared flycatcher is a small, hole‐nesting, migratory passerine, an important model species of population biology and evolutionary ecology (Gustafsson et al., 1995; Török & Tóth, 1988). The study was conducted in a long‐term monitored study population of collared flycatchers located in a forested area near Budapest, Hungary (47°43′N, 19°01′E). This nest box plot system was established in 1982 (Török & Tóth, 1988) and consisted of 658–778 nest boxes during the study period. Collared flycatchers are socially monogamous with biparental care, but genetically polygamous. The species is philopatric (Könczey et al., 1992; Pärt & Gustafsson, 1989), which facilitates the building of a social pedigree. However, the proportion of extra‐pair offspring in the studied population was found to be 17.4%–20.6% (Garamszegi & Møller, 2004; Rosivall et al., 2009), somewhat larger than the 15% reported in a Swedish population (Merilä et al., 1998).

Heritability estimates for the tarsus length of the collared flycatchers have only been estimated in Swedish populations. These estimates range from 0.29 to 0.69 (Kruuk et al., 2001; Merilä, 1997; Silva et al., 2017; Voillemot et al., 2012) and seem relatively stable at shorter time and spatial scales, despite spatial differences in mean phenotypic values (Merilä & Gustafsson, 1996). When investigating the heritability of tarsus length, common environmental effects were found to be substantial (Kruuk et al., 2001; Merilä, 1997) and some effects of cross‐fostering manipulation (Kruuk & Hadfield, 2007) were also detected (but see Alatalo & Lundberg, 1986 for the sister species, the pied flycatcher, *Ficedula hypoleuca*). The heritability of tarsus length did not differ between the sexes and only slightly increased after the exclusion versus inclusion of extra‐pair offspring (Merilä et al., 1998). Additionally, only weak selection was found on tarsus length in Swedish collared flycatchers (Björklund & Gustafsson, 2017; Kruuk et al., 2001; Przybylo et al., 2000). However, selection pressures and evolutionary potential may change between populations, so it is worthwhile to analyze the *Va* in other populations.

### 2.2 Field procedure

Measurements of tarsus length and blood samples for this study were collected between 2003 and 2018 from adult, breeding birds. These birds in the study area were regularly captured with spring traps in their nest boxes when their chicks were 8–10 days old. Then, ringed birds were identified, unringed birds were ringed (with the standard rings of the Hungarian Bird Ringing Centre), and morphological measurements were taken from all individuals, including right tarsus length (from the indentation of the tarsal joint until the base of the fingers bent back) by a caliper with a precision of 0.1 mm. We determined the sex of the birds and the age of the males (i.e. 1 year old or older) based on their plumage (Mullarney et al., 1999). The exact age was only known for locally born birds (hereafter recruits) and males captured for the first time when 1 year old. The exact age of the females born outside the nest boxes and captured for the first time and more than 1‐year‐old non‐recruit males could not be reliably assessed based on plumage traits, so we assigned minimum age to these birds (1 year to females and 2 years to non‐yearling males). Blood samples of a few microliters were taken from the brachial vein and stored in absolute ethanol.

The research area belongs to the Duna‐Ipoly National Park, and the collared flycatcher is a protected species. Therefore, permits for the fieldwork have been provided by the Middle‐Danube‐Valley Inspectorate for Environmental Protection, Nature Conservation and Water Management or the Government Office of Pest county (ref. nos.: DINP 2256‐3/2002, DINP 1931‐2/2003, DINP 2573/2/2004, KTVF/15951/2005, KTVF/22021/2006, KTVF 16360‐2/2007, KTVF 30871‐1/2008, KTVF 43355‐1/2008, KTVF 45116‐2/2011, KTVF 21664‐3/2011, KTVF 12677‐4/2012, KTVF 10949‐8/2013, KTF 11978‐5/2015, PEI/001/1053‐6/2015, PE/EA/101‐8/2018, PE‐06/KTF/8550‐4/2018, and PE‐06/KTF/8550‐5/2018). The capture and the handling of the birds were carried out in a way that minimized welfare impact.

### 2.3 Genetic methods

DNA were extracted from blood samples using a DNeasy Tissue Kit (Qiagen), and concentration was assessed by using a Qubit Fluorometer (Life Technologies).

A paired‐end library (2 μg of genomic DNA per sample, digested with PstI) was prepared following the manufacturer's specifications at CNAG‐CRG (National Genome Analyses Centre, Barcelona, Spain) and sequenced on an Illumina HiSeq2000 v4 with 2 × 125 bp reads at a depth of approximately 10×. Library and sequencing conditions had previously been optimized in a pilot run, in which four samples (8 μg DNA) were digested with multiple restriction enzymes (PstI, ApeKI). To evaluate the reliability of the sequencing process and optimize loci assembly, 20 individuals were included as duplicates.

Raw sequences were inspected with FASTQC (Andrews, 2010) for quality control, demultiplexed, and trimmed to remove the Illumina adapter and reads containing at least a single base, with a Phred quality score of less than 10 (Toonen et al., 2013). We also removed sequences with a Phred quality score of less than 20 in more than 5% of the bases. For each individual sample, FASTQs were aligned against the falbicollis.FicAlb1.5 reference genome using bwa‐mem in BWA v0.7.8 (Li & Durbin, 2009). Aligned BAM files were post‐processed using SAMTOOLS v1.0 (Danecek et al., 2021) and PICARD v1.110 (2019) for respectively filling in mate coordinates and insert size fields and adding read groups. Finally, an indel realignment was performed using RealignerTargetCreator and IndelRealigner from GATK v3.6 (O'Connor & van der Auwera, 2020). The variant calling was obtained using UnifiedGenotyper (GATK v3.6) with all the BAM files from all the samples as input. Resulting SNPs were filtered, and we kept the variants in which ≥1 samples with a genotype not equal to ./. or 0/0 were supported by a depth ≥ 10, GQ ≥ 0 with ≥2 reads different to the reference allele and a frequency ≥0.05.

SNPs were filtered using PLINK 1.07 (Purcell et al., 2007). We removed loci that were not in Hardy–Weinberg equilibrium (*p*‐value < .001), were located on sex chromosomes, and those with a minor allele frequency below 0.05 or a maximum missing rate per SNP of 0.1. Individuals considered in further analyses had no more than 5% missing data. PLINK 1.07 was also used to build a relatedness matrix based on IBS (Identity by State) alleles. The final similarity matrix (hereafter G) was based on 188,231 SNPs and contained 704 individuals.

### 2.4 Social pedigree

Almost all birds breeding in the nest boxes in our study area are captured, and all nestlings are ringed, which allowed us to create a social pedigree. In the rare cases when one or both parents of the clutches were unknown, we inserted common dummy parents for the chicks (assigning a random code as a parent for all sibling chicks) to retain as much information as possible. Chicks in cross‐foster experiments were assigned to their original parents. Cross‐foster manipulation was conducted at 8% of the nests, and the number of chicks involved was 254 (6%) in our dataset. The inclusion of these cross‐fostered chicks is expected to cause only a minor bias, if any (Alatalo & Lundberg, 1986; Kruuk & Hadfield, 2007). The pedigree contained 4521 individuals, 1297 maternities, 1234 paternities, and 384 full siblings. The mean pairwise relatedness was 0.0002, and the maximum pedigree depth was eight generations.

For the analyses using P, the pedigree matrix was trimmed to contain only individuals with genetic marker data. The social pedigree trimmed to the individual with genetic data contained 807 individuals, 212 maternities, 204 paternities, and 44 full siblings. The mean pairwise relatedness was 0.0011, and the maximum pedigree depth was seven generations. Note that in this data subset, 47 birds (7%) were born in cross‐fostered nests, which is almost the same ratio as in the whole dataset.

We could also use a genetically derived pedigree besides the P and the G, but the structure of our data (we had relatively few related birds) prevented this. In our data, only 16%–17% of the parents (fathers and mothers, respectively) were genotyped, and we only know the exact age and thus the birth year for 49% of the birds, which could result in low reliability when deriving the pedigree from genetic data alone (Huisman, 2017).

### 2.5 Statistical analyses

The whole dataset contained 7604 tarsus length measurements from 4381 individuals (2340 females and 2041 males). The number of measurements collected in 1 year varied between 185 and 755, with a mean of 475.25 ± 196.12. In the data subset containing only individuals with genetic data, we had 1630 measurements from 704 individuals (387 females and 317 males). For the data subset, 25 to 209 measurements were collected yearly (mean = 101.63 ± 61.21). The measurements were taken by eight trained researchers (the data subset only by five of them).

Briefly, we first compared the two relatedness matrices (P and G) and assessed the rate of EPP in our sample. Then, we assessed *Va* with animal models using the two different matrices.

**FIGURE 1:**
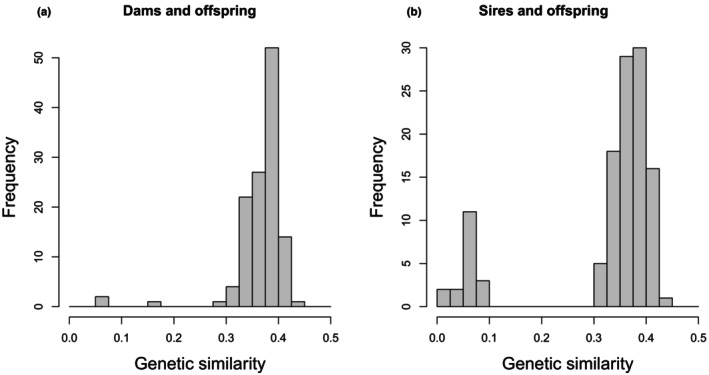
Distribution of the genetic similarity values of (a) dams and (b) sires with their offspring according to the social pedigree.

We compared the two relationship matrices with Mantel tests from the ‘ecodist’ R package (Goslee & Urban, 2007). For assessing the rate of EPP, we identified father–offspring relationships for which genetic data were available for both father and offspring. We assumed that the social father (captured while feeding the nestlings) was indeed the actual genetic father if their genetic similarity was higher than 0.3, and the relationship was classified as EPP if the value was below 0.1 (there was no intermediate value, see Figure 1). The relatedness thresholds were validated by examining also the distribution of the genetic similarity values between mothers and offspring (see Figure 1). Additionally, our thresholds are similar to those of another study on wild birds (Perrier et al., 2018).

We used the animal model framework to decompose the phenotypic variance into genetic and environmental components (Kruuk, 2004; Wilson et al., 2010). Animal models capable of separating variances of different origins (e.g., additive genetic, maternal, common environment, and permanent environmental effects) are excellent tools for estimating the *Va* of traits in wild populations (de Villemereuil et al., 2013; Kruuk, 2004; Postma, 2014; see Equation 1). The model formula was

(1) $$y=\mu +\mathrm{Xb}+Z_{a}a+\mathrm{Zu}+e,$$

where **y** is the vector of the phenotypes, μ is the population intercept, **Xb** is for the fixed effects, **Zu** is for the random effects (e.g., permanent environment), from which **Z**_**a**_ **a**, the additive genetic effect of the individuals, was separated, and **e** is the vector of residuals. **X** is the design matrix for fixed effects, **Z** is the incidence matrix for the random effects, and **b** and **u** are the vectors for the fitted fixed and random effect estimates, respectively. Random effects are drawn from normal distributions with 0 mean and variance estimated from the data.

We used the ‘brms’ R package to fit Bayesian regression models (Bürkner, 2017, 2018). We used default, weakly informative priors (improper flat priors for population‐level effects and half‐Student‐t priors for group‐level effects). We also repeated the analysis with other priors (with different standard deviations and distribution, e.g., Cauchy distribution for group‐level effects) to check whether the results depended on prior choice, but the results remained qualitatively unchanged (not shown). The models ran for 20,000 iterations, with a burn‐in of the first 4000 samples. The trace and distribution of all variables were checked visually, and we also checked mixing and convergence with the potential scale reduction factor (Ȓ; Gelman & Rubin, 1992). Leave‐one‐out cross‐validation was also calculated with the ‘loo’ function. Heritability was calculated as the posterior mean of the ratio of *Va* and the sum of all the variance components, with 95% credible intervals. We also calculated the additive genetic coefficient by dividing the *Va* estimate by the mean of tarsus length. The additive genetic coefficient is considered to reflect the long‐term evolutionary potential of the trait (Hansen et al., 2011; Hansen & Pélabon, 2021; Visscher et al., 2008). Finally, 95% credible intervals were also calculated for these measures from the posterior distribution.

Animal models were built for the subset of birds with genetic data including all repeated measurements. Including repeated measurements in an animal model allows the differentiation of additive genetic and permanent environmental effects, the latter being fixed differences between individuals due to environmental and/or non‐additive genetic effects (Kruuk, 2004; Wilson et al., 2010). In our case, including repeated measurements also facilitated the control of potential confounding factors, such as the effect of the measurer. We included individual relatedness as a random effect in all of the models, where identity was connected to one of the two relationship matrices to estimate additive genetic effects, while another random effect was included for individual identity to estimate permanent environmental effects. In the original models, the control effects were the fixed effects of sex, minimum age (minimum known age at the time of capture), measurer, and the random effect of the year of measurement. Sex was also included as a control variable because the tarsus length of the collared flycatcher was found to differ between the sexes in some studies (Przybylo et al., 2000), although not in others (Merilä et al., 1998; Voillemot et al., 2012). However, in our models, the effect of age and sex and the among‐year variance were negligible. As the other variance estimates were nearly the same, we show the models with only the measurer as a control variable in the main text and the full models in the Appendix (Table S1). The reason for this choice is that we intended to retain the natural sources of variation in the phenotypic variance that would decrease after the inclusion of fixed effects, but on the other hand, we wanted to control for the effect of the measurer that could artificially increase the phenotypic variance (de Villemereuil et al., 2018).We also checked that the additive variance components estimated separately for the sexes were the same (see Table S2).

Additionally, we repeated the analyses with P based on a pedigree without the inserted dummy parents, and the results remained qualitatively unchanged (see Table S3 in the Appendix). Finally, to check whether the subsample of birds with genetic data is a representative sample from the population, we repeated the analysis with P including all available measurements of tarsus length from the study years in the animal models. Under this approach, the heritability estimates were very similar (see Table S4). We originally planned to conduct an analysis on recruit birds calculating also maternal effects, but this analysis was not feasible due to the very few siblings (268 offspring from 238 mothers) in this data subset.

All statistical analyses were performed in the R 3.6.1 statistical environment (R Core Team, 2019).

## 3 RESULTS

### 3.1 Comparison between the relationship matrices

**FIGURE 2:**
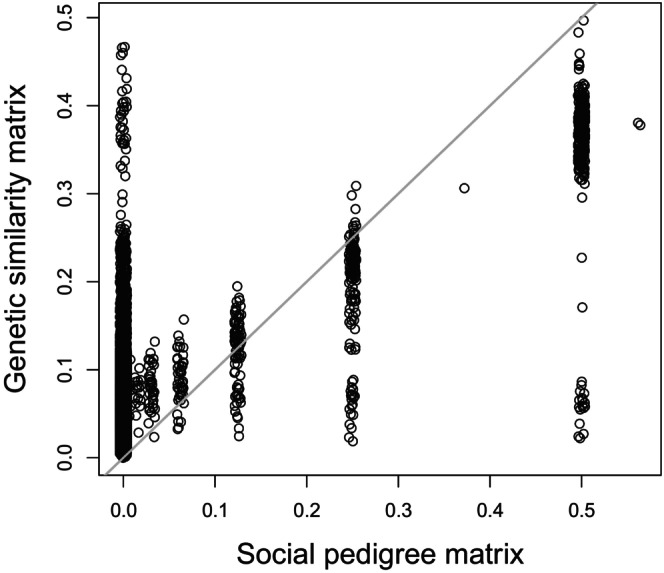
Relationship between the relatedness values of the two relationship matrices used in the study.

The Mantel test between the G and the P yielded a correlation of 0.45 (confidence interval (CI): 0.43–0.46, Figure 2).

By comparing father–offspring relationships in the social pedigree and the genetic similarity matrix, we found 17.54% of EPP (20 EPP out of 114 relationships where both the fathers and offspring had genetic data). Combined with previous results from a paternity analysis (Rosivall et al., 2009) the rate of EPP became 19.23% (38 EPP from 182 father–offspring relationships).

### 3.2 Descriptive statistics of tarsus length

The mean tarsus length in the whole dataset was 17.48 ± 0.54 mm. The mean tarsus length of females was 17.51 ± 0.54 mm, and that of males was 17.46 ± 0.53 mm. For the subset of birds with genetic data, the mean tarsus was 17.46 ± 0.53 mm (for females: 17.46 ± 0.52 mm and for males: 17.45 ± 0.53 mm). Thus, there seems to be no sexual size dimorphism in our present samples.

### 3.3 Results of the animal models

The effect of some of the measurers was significant in all models (see Table 1).

**TABLE 1:** Posterior means for the fixed effects, variance components, heritability, and additive genetic coefficient estimates with 95% credible intervals from the animal models using the two relationship matrices (social pedigree (P) and genetic similarity matrix (G)).

	P	G
Fixed effects		
Measurer2	0.50 (0.05, 0.95)	0.51 (0.07, 0.95)
Measurer3	3.90 (3.55, 4.25)	3.91 (3.56, 4.25)
Measurer4	0.20 (−0.16, 0.56)	0.21 (−0.15, 0.57)
Measurer5	−0.01 (−0.77, 0.75)	−0.01 (−0.77, 0.75)
Random effects		
Additive genetic	13.50 (6.93, 19.90)	20.60 (13.79, 24.97)
Permanent environment	8.22 (2.88, 14.41)	2.22 (0.003, 8.08)
Residual	3.89 (3.55, 4.26)	3.89 (3.56, 4.26)
Heritability	0.53 (0.28, 0.74)	0.77 (0.54, 0.86)
Genetic coefficient	0.08 (0.04, 0.11)	0.12 (0.08, 0.14)

*Note*: Number of observations: 1626, number of individuals: 702.

**FIGURE 3:**
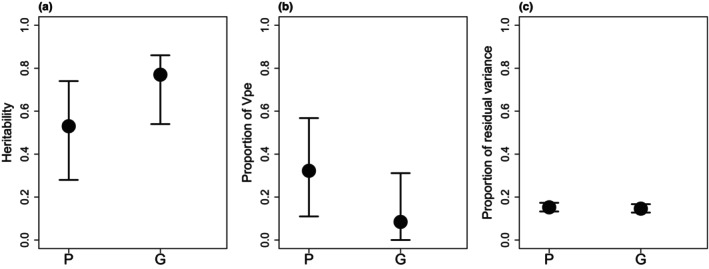
Heritability and proportion of permanent environmental variance (Vpe) and residual variance relative to the total phenotypic variance for the tarsus length of collared flycatchers with 95% credible intervals from the animal models using social pedigree (P) and genetic similarity (G) to assess relatedness among the individuals.

*Va* and heritability estimates were significantly higher with the G than with the P based on the non‐overlapping 95% credible intervals (Table 1, Figure 3). The permanent environmental variance estimates showed an opposite trend to *Va* estimates: the permanent environmental variance estimate from the model using P was significantly higher than the estimates using G. Estimates of residual variance were nearly identical in both models.

## 4 DISCUSSION

Using tarsus length, a structural trait indicative of body size, we have shown that the approach used to estimate relatedness among individuals might impact the estimated *Va* of a trait. While *Va* for this structural trait was high regardless of the matrices used, the correlation between the matrices was moderate, and estimates using the social pedigree and the genetic similarity matrix were significantly different. This potential bias should be addressed when *Va* is assessed in wild populations. Our results when compared to those of other studies also reveal some among‐population differences in the heritability of tarsus length.

Heritability estimates were significantly lower when relatedness was assessed based on the social pedigree matrix instead of genetic data. The lower heritability estimates were due to a decrease in *Va* and a parallel increase in permanent environmental variance in the models using social pedigree instead of genetic similarity matrix, while residual variance estimates remained unchanged. Consistent with the difference in the *Va* estimates, the correlation between the relatedness matrices based on genetic similarity and social pedigree was relatively low. Thus, it seems that the social pedigree reflected relatedness between the individuals less reliably, and consequently, some variance that is part of the additive genetic variance according to the models with the genetic similarity matrix became allocated to the permanent environmental variance. Several nonmutually exclusive factors could explain these differences, such as (i) a relatively high rate of EPP in our population (around 20% (this study; Garamszegi & Møller, 2004; Rosivall et al., 2009), which otherwise is in the range of that found in other passerines with social monogamy (Canal et al., 2011; Charmantier & Réale, 2005; Firth et al., 2015)), (ii) of the presence of distant relatives that are unconnected in our social pedigree (although the probability of distant relatives breeding close to each other is low in our population), and (iii) the existence of numerous zeros in the social pedigree matrix as opposed to the absence of these values in the genetic matrix. These causes are related to the fact that the genomic similarity matrix reflects realized relatedness instead of expected relatedness (Visscher et al., 2006). Among these factors, the influence of EPP on heritability has received particular attention. For example, in earlier studies, using a corrected pedigree led to an increase in heritability estimates (Keller et al., 2001). If the gain/loss of EPP is associated with tarsus length, the biasing effect of this phenomenon could be more serious (Firth et al., 2015). This is a possible scenario, as males with longer tarsi had more extra‐pair offspring and thus greater reproductive success in tree swallows (*Tachycineta bicolor*) (Lessard et al., 2014) and pied flycatchers (Canal et al., 2011) and females with shorter tarsi had an increased probability of extra‐pair copulation in our study species (Rosivall et al., 2009). Thus, the bias of heritability estimates obtained with social pedigrees may be lower in populations with low levels of EPP and probably also when the relatedness among the individuals is high. Other studies comparing heritability estimates using genetic and pedigree data from wild populations of other species show inconsistent results. A study with blue tits (*Cyanistes caeruleus*) found slightly lower values based on social pedigree than based on genetic data (Perrier et al., 2018). In contrast, estimates for the heritability of wing length in great tits (*Parus major*) and for body size in soay sheep (*Ovis aries*) were very similar when using pedigree versus marker‐derived estimates (Bérénos et al., 2014; Robinson et al., 2013). The fact that we found differences between the different approaches, while other studies did not, may be explained again by the higher rate of EPP in our study population or by differences in population structure between the species (Quinn et al., 2006), which may have caused a greater divergence in the relatedness based on social pedigree or genetic data. Additionally, the study on soay sheep used microsatellite data to determine paternity (and not a solely social pedigree), that could explain the small differences between the different methods (Bérénos et al., 2014). Another study on the laying date of great tits found slightly larger heritability estimates with pedigree than with the genetic similarity matrix, and this was explained by the correct allocation of environment‐induced similarity between relatives by the methods using the genetic similarity matrix (Gienapp et al., 2019). In contrast, we showed higher heritability with the genetic similarity matrix. However, a limitation of our study is that, due to lack of data, we could not control for maternal and common environmental effects. These effects, if present and do not controlled for, could inflate our heritability estimates (Wilson et al., 2010). Due to the low number of siblings in our data set, the aforementioned effects may have low influence and rather the inaccuracy of the social pedigree data caused the lower heritability estimates obtained with it. Nevertheless, our results highlight a potential problem in *Va* estimation when relatedness is assessed based on field‐observed pedigrees.

Although our results suggest that better estimates of heritability can be obtained with a genetic relatedness matrix, the benefits of the different methods can be system‐specific. It may be easier to collect high‐quality data for social pedigree in some systems, e.g., in species where breeding is easily observed such as birds nesting in artificial nest boxes (with a low level of EPP) or mammals breeding in burrows (Pemberton, 2008), and also when philopatry is high and immigration is low; thus, the relatedness between individuals in the population is relatively high (Kruuk & Hadfield, 2007). However, it can be difficult to obtain a reliable pedigree in other systems, for instance, in species in which observing paternity is difficult, e.g., because they are promiscuous (Garant et al., 2003; Pemberton et al., 1999), in species that are very rare, relatively long‐lived with a long generation time and also when the variance in relatedness is low between the individuals (Kruuk & Hadfield, 2007; Quinn et al., 2006). In the latter cases, collecting genetic data can be a good solution, but again, in certain populations, collecting DNA could be hindered due to ethical concerns or financial constraints, e.g., when the population is large and many individuals have to be sampled (Galla et al., 2022; Pemberton, 2008). Building social pedigrees maybe especially useful in conservation, where reliable data for this are easily collected from managed or captive population and in parallel, other relevant data such as demographic data, fitness measures, and data on symptoms reflecting inbreeding depression can be obtained (Galla et al., 2022). There are also very valuable long‐term datasets where genetic data are not available and relatedness can only be assessed based on social pedigrees. Thus, the decision to use social pedigree or genetic data to assess relatedness between the individuals should be made considering the characteristics of the study system and the time and financial budget available for the study.

The heritability estimates with the matrices derived from the social pedigree (0.53, 0.66) were comparable to most of those previously reported for the study species, while the heritability obtained using the genetic similarity matrix (0.77) was higher than that of these previous estimates (Merilä & Gustafsson, 1996; Silva et al., 2017) (see also Table 2). On the whole, our results are comparable to those of the heritability estimates for tarsus length reported in other passerines such as great tits (Husby et al., 2011; Van Noordwijk et al., 1988), blue tits (Perrier et al., 2018), and great reed warblers (*Acrocephalus arundinaceus*) (Åkesson et al., 2008), but higher than those found in house martins (*Delichon urbica*) (Christe et al., 2000). Body size also has high heritability in various other taxa from insects to humans (Bérénos et al., 2014; Visscher et al., 2006; Walsh et al., 2020; Zaitlen et al., 2013) (see also Table 2). It should be noted that apart from population differences in additive genetic or environmental variance components (Husby et al., 2011), differences between the studies on the collared flycatcher could arise from different methodological approaches (parent–offspring regression vs. animal model) or sample sizes (de Villemereuil et al., 2013; Quinn et al., 2006), hindering the comparisons between studies. However, one study with a similar methodology and sample size revealed much lower heritability (0.289; Silva et al., 2017) than ours, indicating potential population differences in the tarsus length heritability of collared flycatchers. Although our work and that of Silva et al. (2017) show similar *Va* estimates, the total phenotypic variance was larger in the Swedish population, indicating that probably the greater environmental variance in this latter population (e.g., maybe due to the harsher climate or the longer migration to the breeding grounds) caused the difference. However, further research, i.e., with heritability estimates from multiple populations using the same methodology, is necessary to provide insights into this issue.

**TABLE 2:** Details of the referenced studies investigating the heritability of body size.

Study	Species	Method	Sample size^a^	Heritability estimate^b^	Trait
Birds					
Åkesson et al. (2008)	*Acrocephalus arundinaceus*	Partly genetically derived pedigree, including repeated measurements	456	0.711 ± 0.084	Tarsus length
	*Acrocephalus arundinaceus*	Partly genetically derived pedigree, including the mean of the measurements	456	0.727 ± 0.112	Tarsus length
Perrier et al. (2018)	*Cyanistes caeruleus*	Genome‐wide relatedness matrix, including repeated measurements	489	0.79 (0.65–0.87)	Tarsus length
	*Cyanistes caeruleus*	Corrected pedigree, including repeated measurements	489	0.67 (0.49–0.80)	Tarsus length
	*Cyanistes caeruleus*	Social pedigree, including repeated measurements	494	0.62 (0.46–0.73)	Tarsus length
Christe et al. (2000)	*Delichon urbica*	General linear model, estimation of additive genetic effects from the nest of the origin effect	34 pairs of nests, 144	0.079	Tarsus length
Merilä and Gustafsson (1996)	*Ficedula albicollis*	ANCOVA (equivalent with parent–offspring regression)	51–106 (7 consecutive years)	0.25 ± 0.14–0.67 ± 0.14	Tarsus length
Kruuk et al. (2001)	*Ficedula albicollis*	Pedigree	23,000	0.35	Tarsus length
Voillemot et al. (2012)	*Ficedula albicollis*	General linear model, estimation of additive genetic effects from the nest of the origin effect	359	0.39	Tarsus length
Silva et al. (2017)	*Ficedula albicollis*	Genomic relationship matrix, including repeated measurements	798	0.289 ± 0.07	Tarsus length
	*Ficedula albicollis*	Genomic relationship matrix, including the mean of the measurements	798	0.576 ± 0.079	Tarsus length
Van Noordwijk et al. (1988)	*Parus major*	Parent offspring regression, using nest means	59 broods	0.52 ± 0.13	Tarsus length
Husby et al. (2011)	*Parus major*	Social pedigree, including repeated measurements	3283, 966, 3191 (three populations)	0.525 ± 0.039, 0.317 ± 0.058, 0.214 ± 0.023	Tarsus length
Silva et al. (2017)	*Passer domesticus*	Genomic relationship matrix, including repeated measurements	1443	0.415 ± 0.042	Tarsus length
	*Passer domesticus*	Genomic relationship matrix, including the mean of the measurements	1443	0.399 ± 0.041	Tarsus length
Mammals					
Bérénos et al. (2014)	*Ovis aries*	Pedigree (maternities based on observation, paternities based on genetic data)	816	0.468 ± 0.065	Adult hindleg^c^
	*Ovis aries*	Genetically derived pedigree	891	0.458 ± 0.058	Adult hindleg^b^
	*Ovis aries*	Genomic relatedness matrix	867	0.441 ± 0.051	Adult hindleg^b^
Visscher et al. (2006)	*Homo sapiens*	Genome‐wide IBD sharing	2444 sib pairs	0.80 (0.43–0.86)	Adult height
Zaitlen et al. (2013)	*Homo sapiens*	Genetic similarity matrix	20,000	0.693 ± 0.016	Body height
Insects					
Walsh et al. (2020)	*Monomorium pharaonis*	Pedigree	243 colonies	Worker: 0.34, gyne: 0.46, male: 0.53	Body mass

*Note*: The list is not comprehensive; it only illustrates the range of values and the variety of groups in which this topic was investigated.

^a^ Number of individuals if not otherwise indicated.

^b^ Heritability estimates are reported together with ± standard deviation or with confidence intervals in brackets.

^c^ We selected to show here the trait with the highest sample size, but other proxies of body size can be seen in the reference (including repeated measurements).

The *Va* found in tarsus length can have evolutionary significance. This is corroborated by the additive genetic coefficient of variation estimates which are considered to reflect evolvability (Houle, 1992). These were between 0.08 and 0.12 in our study and are somewhat greater than the values found for morphological traits in house martins (0–0.07) (Christe et al., 2000), but within the range of values for various morphological and life history traits in *Drosophila melanogaster* (0.02–0.12) (Houle, 1992). Studies on selection differentials of tarsus length in the collared and pied flycatchers in Swedish populations suggest that the trait is well‐adapted (Alatalo & Lundberg, 1986; Björklund & Gustafsson, 2017; Kruuk et al., 2001; Przybylo et al., 2000) and the environment of the studied populations is relatively stable (Björklund & Gustafsson, 2017). However, as selection pressures could differ between populations (Husby et al., 2011), more studies are needed to confirm the generality of these findings. Nevertheless, *Va* in tarsus length, which can contribute to population persistence under adverse conditions such as those associated with the ongoing environmental change, is still present in all the investigated populations.

Taken together, we report a marked difference between *Va* and heritability values estimated based on social pedigree versus genetic similarity. Our results suggest that estimates based on social pedigree could be downwardly biased, which is an issue that should be considered especially in studies conducted in the wild. Additionally, we found high *Va* and heritability estimates for tarsus length in a migratory passerine, revealing a critical trait's capacity to respond to selection.

## AUTHOR CONTRIBUTIONS

**Mónika Jablonszky:** Conceptualization (equal); data curation (equal); formal analysis (lead); investigation (equal); visualization (lead); writing – original draft (lead). **David Canal:** Formal analysis (supporting); writing – review and editing (equal). **Gergely Hegyi:** Investigation (equal); writing – review and editing (equal). **Márton Herényi:** Data curation (equal); investigation (equal); writing – review and editing (equal). **Miklós Laczi:** Data curation (equal); investigation (equal); writing – review and editing (equal). **Gábor Markó:** Investigation (equal); writing – review and editing (equal). **Gergely Nagy:** Data curation (equal); investigation (equal); writing – review and editing (equal). **Balázs Rosivall:** Investigation (equal); writing – review and editing (equal). **Eszter Szöllősi:** Investigation (equal); writing – review and editing (equal). **János Török:** Investigation (equal); writing – review and editing (equal). **László Zsolt Garamszegi:** Conceptualization (equal); formal analysis (supporting); funding acquisition (equal); investigation (equal); writing – review and editing (equal).

## CONFLICT OF INTEREST STATEMENT

The authors declare no conflict of interest.

## Supporting information


Tables S1–S4.

## Data Availability

The data used to obtain the results was uploaded to Figshare: https://doi.org/10.6184/m9.figshare.25052864.v1. (Jablonszky et al., 2024).
