# Supplementary material for: The estimation of additive genetic variance of body size in a wild passerine is sensitive to the method used to estimate relatedness among the individuals
Source: Ecol Evol. 2024 Feb 13;14(2):e10981. doi: 10.1002/ece3.10981 (PMC10862163; doi:10.1002/ece3.10981)
Supplement: Supplementary file 1 — Tables S1–S4. [file ECE3-14-e10981-s001.docx]

Appendix

Table S1: Results from the animal models with the two relationship matrices (social pedigree (P) and genetic similarity matrix (G)) using all control variables. Posterior means and 95% credible intervals for the fixed and random effects, heritability and additive genetic coefficient estimates are displayed. Number of observations: 1626, number of individuals: 702

|  | P |  | G |
| --- | --- | --- | --- |
| Fixed effects |  |  |  |
| Age | 0.10 (-0.01, 0.21) |  | 0.09 (-0.02, 0.20) |
| Sex (male) | -0.26 (-1.01, 0.47) |  | -0.37 (-1.14, 0.39) |
| Measurer2 | 0.48 (0.05, 0.92) |  | 0.49 (0.05, 0.94) |
| Measurer3 | 3.97 (3.62, 4.31) |  | 3.97 (3.62, 4.31) |
| Measurer4 | 0.38 (0.03, 0.74) |  | 0.39 (0.03, 0.74) |
| Measurer5 | -0.30 (-1.06, 0.47) |  | -0.29 (-1.05, 0.47) |
| Random effects |  |  |  |
| Additive genetic | 13.90 (6.88, 20.66) |  | 21.68 (14.42, 25.99) |
| Permanent environment | 8.50 (2.91, 15.14) |  | 1.91 (0.002, 8.20) |
| Year of measurement | 0.54 (0.18, 1.33) |  | 0.54 (0.17, 1.33) |
| Residual | 3.56 (3.24, 3.90) |  | 3.56 (3.24, 3.90) |
| Heritability | 0. 52 (0.27, 0.74) |  | 0.78 (0.54, 0.87) |
| Genetic coefficient | 0.08 (0.04, 0.12) |  | 0.12 (0.09, 0.16) |

Table S2: Additive genetic variance and heritability estimates from the animal models estimating these components separately for the two sexes. Only the measurer was used as a control variable

|  | Additive genetic variance | | Heritability | |
| --- | --- | --- | --- | --- |
|  | Females | Males | Females | Males |
| P | 12.72 (5.16, 19.86) | 12.90 (5.51, 20.11) | 0.49 (0.21, 0.72) | 0.50 (0.23, 0.72) |
| G | 21.22 (13.80, 26.54) | 20.27 (13.53, 25.56) | 0.78 (0.55, 0.87) | 0.77 (0.54, 0.86) |

Table S3: Results from the animal models using a social pedigree without dummy parents. Posterior means and 95% credible intervals for the fixed and random effects, heritability and additive genetic coefficient estimates are displayed. Restricted models only included the measurer as a control effect, while full models included also the fixed effects of sex, age and the random effect of year of measurement. Number of observations: 1626, number of individuals: 702

|  | P | |  | |
| --- | --- | --- | --- | --- |
|  | Restricted | Full |  |  |
| Fixed effects |  |  |  |  |
| Age |  | 0.10 (-0.01, 0.21) |  |  |
| Sex (male) |  | -0.27 (-1.01, 0.47) |  |  |
| Measurer2 | 0.50 (0.06, 0.94) | 0.48 (0.05, 0.92) |  |  |
| Measurer3 | 3.90 (3.55, 4.25) | 3.97 (3.62, 4.32) |  |  |
| Measurer4 | 0.20 (-0.16, 0.56) | 0.38 (0.03, 0.74) |  |  |
| Measurer5 | -0.02 (-0.78, 0.73) | -0.29 (-1.06, 0.47) |  |  |
| Random effects |  |  |  |  |
| Additive genetic | 13.62 (6.99, 20.01) | 14.03 (7.21, 20.71) |  |  |
| Permanent environment | 8.13 (2.93, 14.47) | 8.37 (2.98, 14.72) |  |  |
| Year of measurement |  | 0.53 (0.18, 1.28) |  |  |
| Residual | 3.89 (3.54, 4.27) | 3.55 (3.24, 3.90) |  |  |
| Heritability | 0.53 (0.28, 0.74) | 0.53 (0.28-0.74) |  |  |
| Genetic coefficient | 0.08 (0.04, 0.11) | 0.08 (0.04, 0.12) |  |  |

Table S4: Results from the animal models with all data on tarsus length included. Posterior means and 95% credible intervals for the fixed and random effects, heritability and additive genetic coefficient estimates are displayed. Number of observations: 7591, number of individuals: 4372

|  | P |  |
| --- | --- | --- |
| Fixed effects |  |  |
| Age | 0.04 (-0.02, 0.10) |  |
| Sex (male) | -0.50 (-0.80, -0.22) |  |
| Measurer2 | 0.69 (0.41, 0.96) |  |
| Measurer3 | 4.05 (3.85, 4.25) |  |
| Measurer4 | 0.89 (0.69, 1.09) |  |
| Measurer5 | -0.47 (-0.90, -0.05) |  |
| Measurer6 | 2.51 (0.06, 4.91) |  |
| Measurer7 | -1.44 (-3.20, 0.27) |  |
| Random effects |  |  |
| Additive genetic | 13.83 (11.81, 15.91) |  |
| Permanent environment | 8.00 (6.35, 9.74) |  |
| Year of measurement | 0.35 (0.14, 0.81) |  |
| Residual | 4.26 (4.06, 4.47) |  |
| Heritability | 0.52 (0.45, 0.59) |  |
| Genetic coefficient | 0.08 (0.07, 0.09) |  |
